# Characterizing the blood microbiota in healthy and febrile domestic cats via 16s rRNA sequencing

**DOI:** 10.1038/s41598-024-61023-4

**Published:** 2024-05-08

**Authors:** Liam Kitson, Anne A. M. J. Becker, Katrin Hartmann, Michèle Bergmann, Paulina Sepulveda-Garcia, Nivia Canales, Ananda Muller

**Affiliations:** 1 Graduate Program, Ross University School of Veterinary Medicine, West Farm, West Indies Saint Kitts and Nevis; 2 One Health Center for Zoonoses and Tropical Veterinary Medicine, Biomedical Sciences Department, Ross University School of Veterinary Medicine, West Farm, West Indies Saint Kitts and Nevis; 3 LMU Small Animal Clinic, Centre for Clinical Veterinary Medicine, LMU Munich, Munich, Germany; 4 Instituto de Medicina Preventiva Veterinaria, Facultad de Ciencias Veterinarias, Universidad Austral de Chile, Valdivia, Chile; 5 Escuela de Graduados, Facultad de Ciencias Veterinarias, Universidad Austral de Chile, Valdivia, Chile; 6 Instituto de Bioquímica y Microbiología, Facultad de Ciencias, Universidad Austral de Chile, Valdivia, Chile

**Keywords:** Feline microbiota, 16S V3-V4 region, Fever, Infectious disease, Bacteria, Microbial communities, Infectious-disease diagnostics

## Abstract

This study aimed to evaluate the blood bacterial microbiota in healthy and febrile cats. High-quality sequencing reads from the 16S rRNA gene variable region V3-V4 were obtained from genomic blood DNA belonging to 145 healthy cats, and 140 febrile cats. Comparisons between the blood microbiota of healthy and febrile cats revealed dominant presence of *Actinobacteria*, followed by *Firmicutes* and *Proteobacteria*, and a lower relative abundance of *Bacteroidetes*. Upon lower taxonomic levels, the bacterial composition was significantly different between healthy and febrile cats. The families Faecalibacterium and Kineothrix (*Firmicutes*), and Phyllobacterium (*Proteobacteria*) experienced increased abundance in febrile samples. Whereas Thioprofundum (*Proteobacteria*) demonstrated a significant decrease in abundance in febrile. The bacterial composition and beta diversity within febrile cats was different according to the affected body system (Oral/GI, systemic, skin, and respiratory) at both family and genus levels. Sex and age were not significant factors affecting the blood microbiota of febrile cats nor healthy ones. Age was different between young adult and mature adult healthy cats. Alpha diversity was unaffected by any factors. Overall, the findings suggest that age, health status and nature of disease are significant factors affecting blood microbiota diversity and composition in cats, but sex is not.

## Introduction

The host-associated microbiome undertakes several essential biological processes and thus it is unsurprising that several disease states are associated with changes in microbiome structure and function, this status is termed “dysbiosis”^1^. Amplicon sequencing, targeting the 16S rRNA gene, is now one of the most widely used and cost-effective high-throughput methods to investigate bacterial community composition and dynamics^2^.

The advances in the field of metagenomics have allowed the characterization of rich and diverse microbial communities in the gut, vagina, skin, and other tissues in humans^3,4^. Early microbiome studies on different body habitats however, neglected to include the respiratory tract and bloodstream^3,5^, due to the longstanding belief that these systems were sterile. The existence of microbial populations in these “classically sterile” locations, is a relatively new concept^6^. In humans, there is mounting evidence that microbial components in blood could play a crucial role in the pathophysiological events leading up to or during diseases^7^. Moreover, evidence supports the existence of a blood microbiome not only in humans^7–9^ but also in various other species including rodents, chickens, cows, and dogs^10–14^.

In cats, the blood microbiome has only been investigated in a very small number (*n* = 6) of healthy kittens (10 weeks old)^15^. Although blood samples presented lower DNA yields and richness when compared to bronchoalveolar, oropharyngeal and fecal samples, detectable levels of bacteria existed in blood samples from the kittens. *Bacteroidetes* and *Proteobacteria* were the predominant phyla in the blood of kittens^15^. In humans, at the phylum level, the blood microbiota was predominated by *Proteobacteria*, *Actinobacteria*, *Firmicutes*, and *Bacteroidetes*^7^. Studies in humans demonstrate notable differences in the composition of the blood microbiota in health versus disease. The circulating blood microbiota has been analyzed in human patients with leukemia, obesity, diabetes, cirrhosis, and cardiovascular diseases^16–20^. In animals, the blood microbiota has been assessed in dogs with bacterial pneumonia^13^, dogs with diarrhea^21^, broilers with osteomyelitis^11^, cows with metritis^12^, goats affected by *Haemonchus contortus*^22^, and pigs with fecal-induced peritonitis^23^.

Therefore, it is likely that in cats, the blood microbiota is also influenced by disease states. However, the existence and composition of such a bacterial community remains largely unexplored in both healthy and diseased cats. Moreover, the current available data is limited to kittens, and considering that host-associated microbiomes have a reciprocal relationship with host age^24^ one could not assume that the composition would be similar in adult cats. To this end, the aims of this study were three-fold, using high-throughput 16S rRNA gene sequencing (1) to describe the feline blood bacterial diversity by sex and across multiple age ranges in healthy cats; (2) to compare the blood microbiota within febrile cats according to their age, sex and their affected body system and (3) to compare the circulating bacterial community composition between healthy and febrile cats.

## Results

Of the 300 samples from cats, 285 had sufficient DNA concentration. All fifteen failed samples were from the healthy group. More specifically, 5 kittens, 7 young adults, and 3 mature adults failed to amplify any product (0 ng/µl) from the V3-V4 region of the 16S rRNA gene. After removal of these samples, the total number of healthy cats by age category used for library construction was 55 kittens, 53 young adults, 27 mature adults and 10 seniors, totaling 145 samples from healthy cats (Table S1).

After filtering out low quality reads, a total of 7,096,894 reads were obtained with 24,901 reads per sample on average. The minimum number of reads was 2473, the maximum number was 82,749 and the median number of reads was 25,161. The sparsity of the data was 0.9886, indicating that most genomic regions had low or no coverage. After sorting the sequences obtained from the high-throughput sequencing into operational taxonomic units (OTUs) using a 97% sequence similarity identity cut-off and removing unclassified sequences at the kingdom level or lower, a total of 3612 OTUs were obtained.

### Diversity and composition of the bacterial blood microbiota in healthy cats

**Figure 1:**
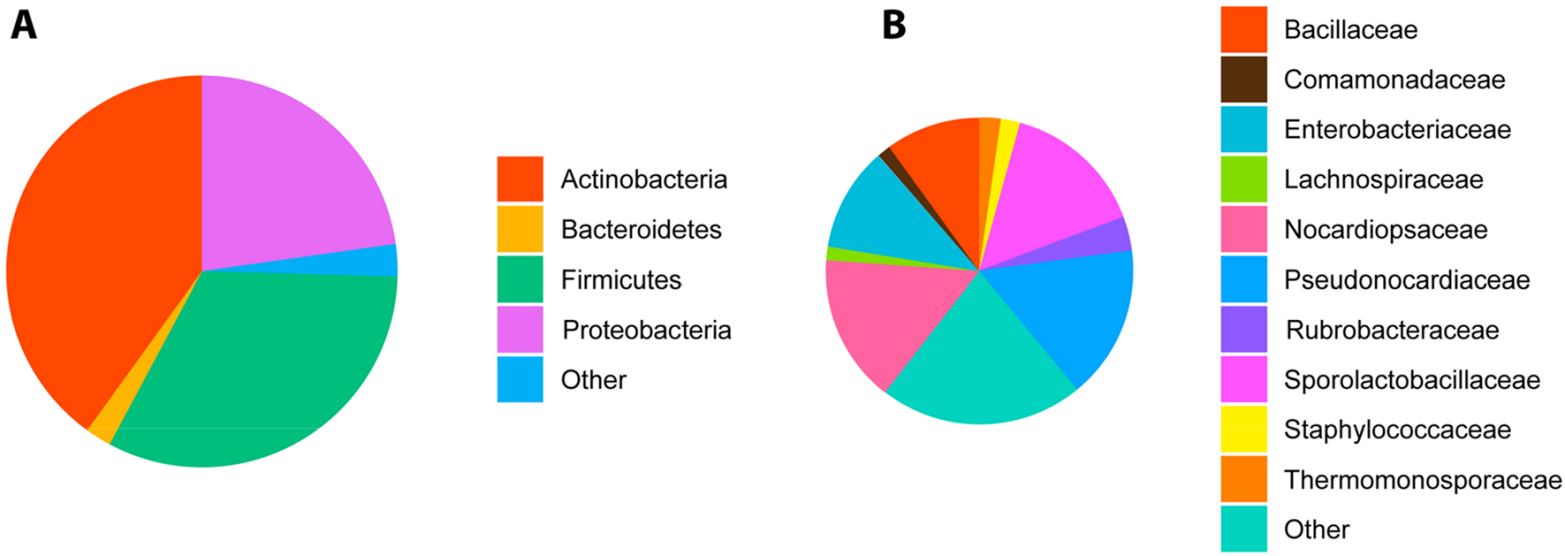
Relative abundance of bacterial phyla over 1% (**A**) and top 10 most abundant families (**B**) in 145 blood samples of healthy domestic cats, determined by 16S rRNA gene sequencing. “Other” groups those phyla with a median relative abundance ≤ 1% or not in the top 10 most abundant families.

Taxonomic assignment of the healthy cats (*n* = 145) revealed a dominant presence, at phylum level, of *Actinobacteria* (39.33% of total reads, ranging from 11.26 to 72.3%) followed by *Firmicutes* (32.02%, [9.61–55.58%]) and *Proteobacteria* (24.01%, [0–78.73%]) and a lower relative abundance of *Bacteroidetes* (2.15%, [0–14.29%]). The remainder of reads could be attributed to marginal phyla (2.49%) (Fig. 1A).

The 10 most abundant families made up 77.05% of the total reads (Fig. 1B). These comprised the *Pseudonocardiaceae* (15.85%, [4.06–32.3%])), *Nocardiopsaceae* (15.26%, [3.77–36.36%]), *SporolactoBacillaceae* (14.73%, [0–30.79%]), *Enterobacteriaceae* (10.77%, [0–54.69%]), *Bacillaceae* (9.84%, [1.96–23.97%]), *Rubrobacteraceae* (3.52%, [0–10.34%]), *Thermomonosporaceae* (2.16%, [0–9.47%]), *Staphylococcaceae* (2.11%, [0–45.35%]), *Comamonadaceae* (1.42%, [0–9.28%]), and *Anaplasmataceae* (1.39%, [0–1.43%]).

Overall, phylogenetic diversity did not significantly differ between sex or age groups in healthy cats, according to the Bray–Curtis beta diversity analysis, as distinct clusters could not be observed (p > 0.05; Fig. S1).

According to the Kruskal–Wallis rank sum test used to compare healthy cats by sex no significant difference in median relative abundance between sexes was observed at the level of phylum or family levels (Fig. S2).

**Figure 2:**
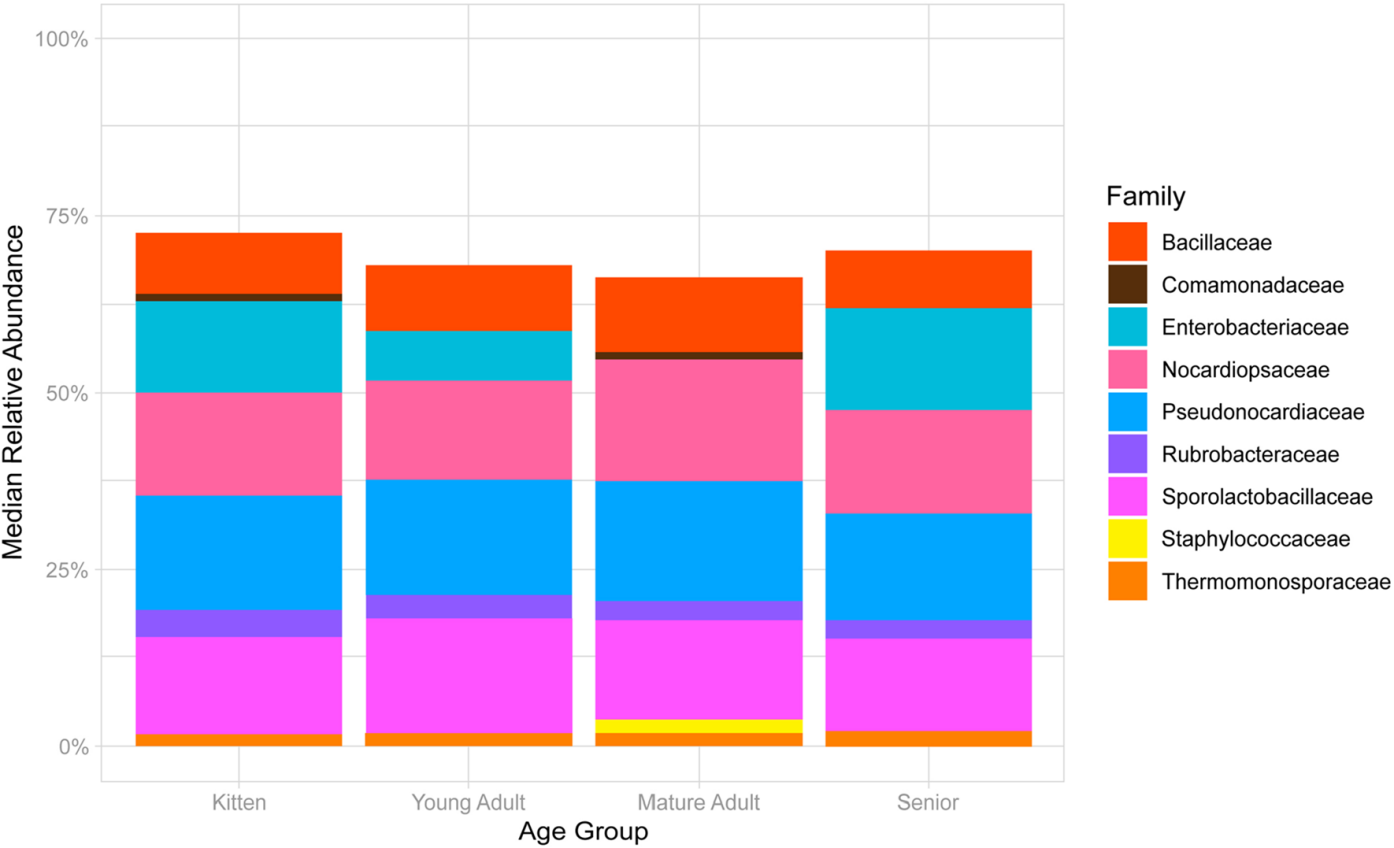
Taxonomic distribution of the healthy feline blood microbiota (*n* = 145) by age group, at the family level. Only families with a median relative abundance > 1% are included in the graph.

There were also no notable compositional differences at the phylum level between age subgroups. However, significant differences of taxonomic distribution were observed, at family level among the four age subgroups within the healthy cat cohort (Kruskal–Wallis, p = 0.0055), particularly between the young adult and mature adult groups (pairwise Wilcoxon, p = 0.0098). Young adults and mature adults were significantly different (Wilcoxon, p < 0.05). The largest differences between the young adult and mature adult cohort were at family taxonomic level, with the increased presence of *Enterobacteriaceae* and decreased presence of *Staphylococcaceace* in the young adults (Fig. 2).

### Diversity and composition of the bacterial blood microbiota in febrile cats

**Figure 3:**
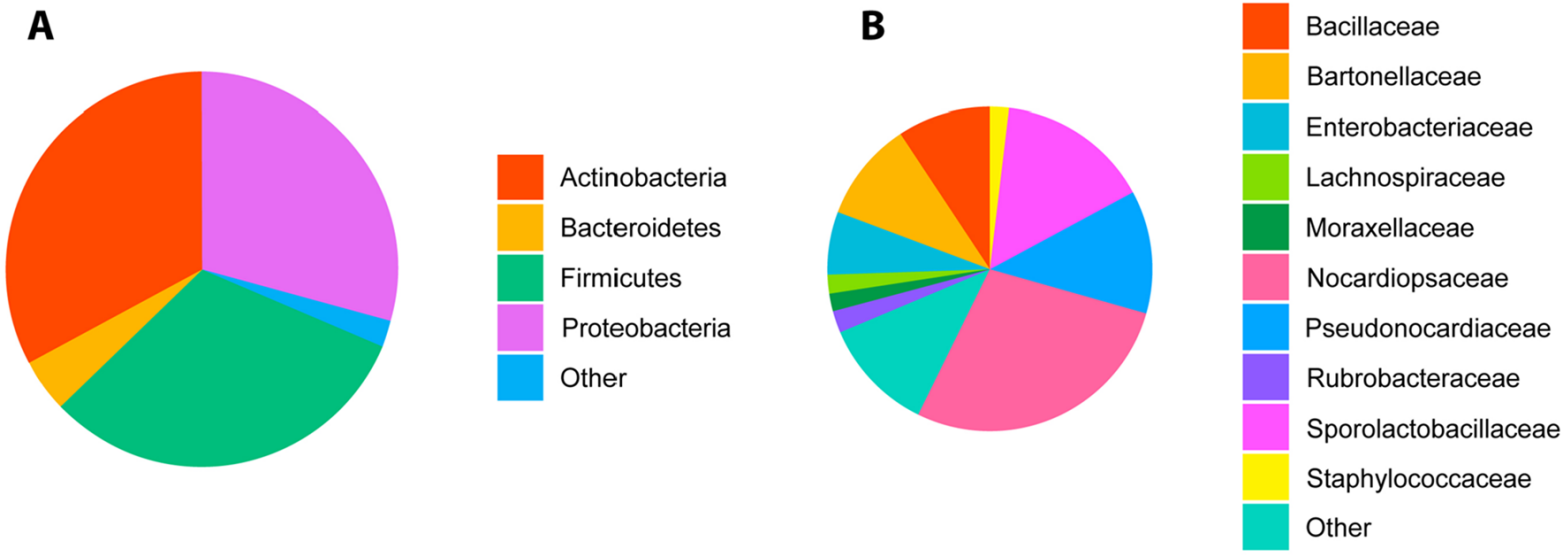
Relative abundance of bacterial phyla over 1% abundance (**A**) and top 10 most abundant families (**B**) in 140 blood samples of febrile domestic cats, determined by 16S rRNA gene sequencing. “Other” groups those phyla with a median relative abundance ≤ 1% or not in the top ten at family level.

Taxonomic assignment of the febrile cats (*n* = 140) at the phylum level revealed a dominant presence of *Actinobacteria* (32.59% of total reads, ranging from 0.5 to 76.2%), *Firmicutes* (31.21% [0.8–89.0%]) and *Proteobacteria* (29.23%, [1.0–97.9%]), and a lower relative abundance of *Bacteroidetes* (4.64%, [0–55.9%]). The remainder of reads could be attributed to marginal phyla (2.34%) (Fig. 3A).

The 10 most abundant families made up 70.13% of the total reads (Fig. 3B). These comprised the *Pseudonocardiaceae* (12.24% [0.1–32.2%]), *SporolactoBacillaceae* (12.19% [0.1–40.2%]), *Nocardiopsaceae* (11.30% [0.1–39.6%]), *Bartonellaceae* (10.00% [0–97.7%]), *Enterobacteriaceae* (6.26% [0–26.0%]), *Bacillaceae* (9.22% [0.4–28.2%]), *Rubrobacteraceae* (2.91% [0–14.0%]), *Moraxellaceae* (2.27% [0–43.4%]), *Staphylococcaceace* (1.96% [0–24.4%]) and *Lachnospiraceae* (1.78%, [0–24.6%]) (Fig. 3B).

**Figure 4:**
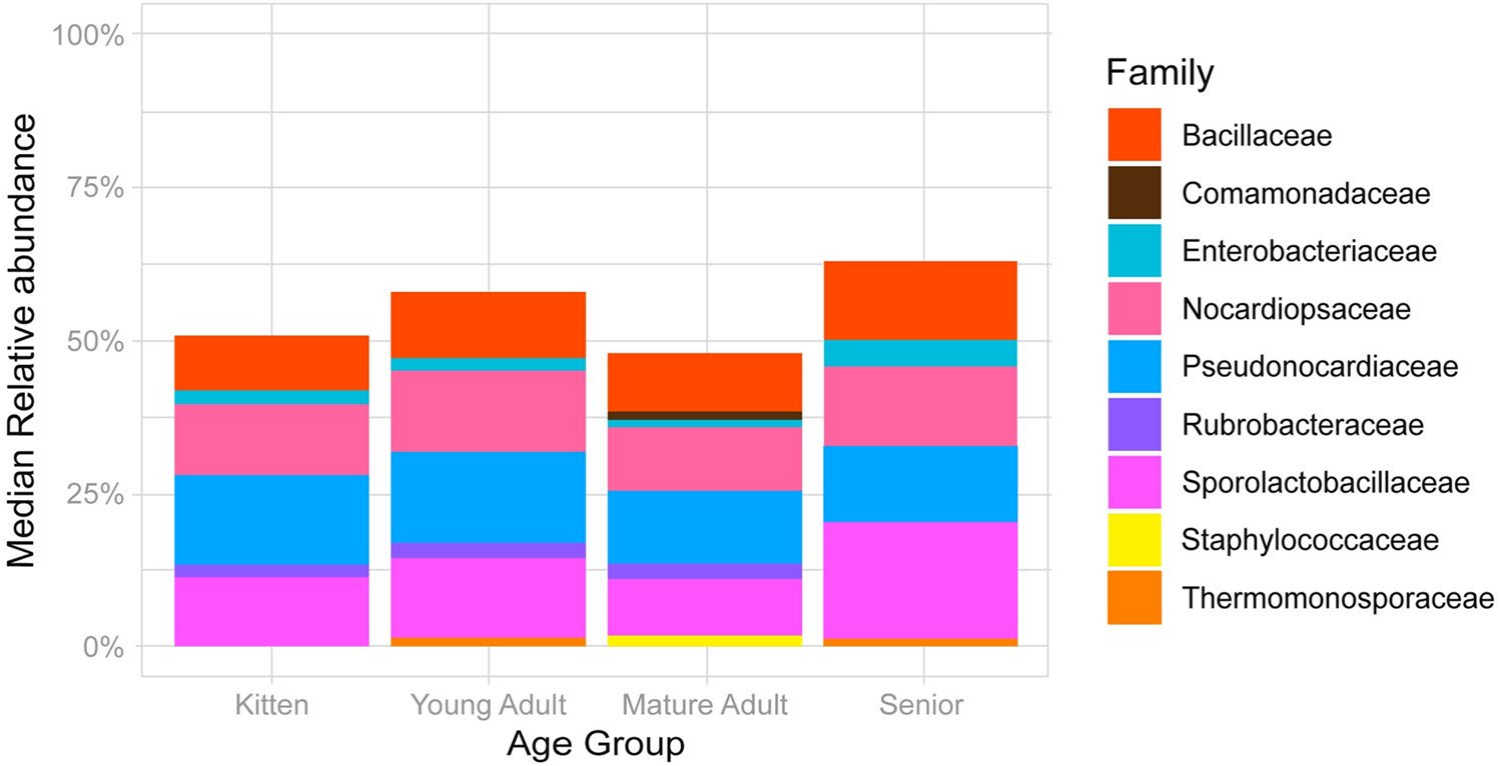
Taxonomic distribution of the febrile feline blood microbiota (*n* = 140) by age group, at family level. Only families with a median relative abundance > 1% are included in the graph.

Assessments of composition and richness by sex did not differ significantly at phylum nor family level. Furthermore, the febrile group demonstrated non-significant differences at the family level by age group (Kruskal–Wallis, p = 0.053; Pairwise Wilcoxon, p > 0.05) (Fig. 4).

**Figure 5:**
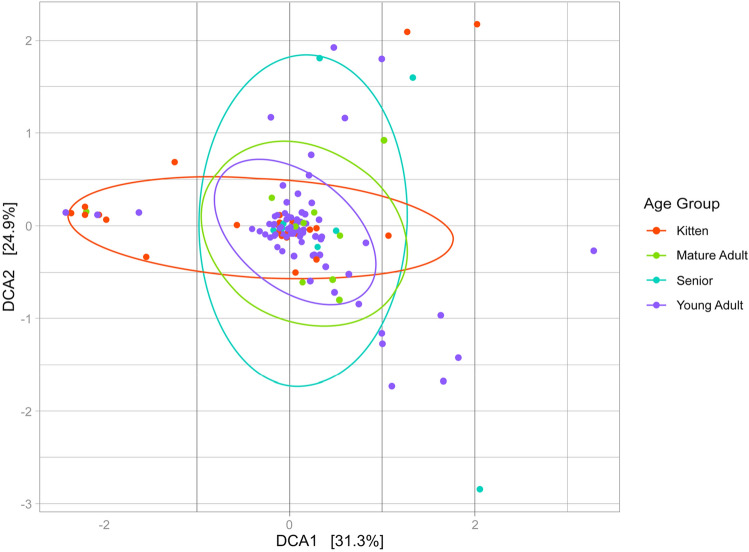
Principal component analysis (PCoA) of the blood microbiota of febrile cats excluding one outlier (*n* = 139), based on Bray–Curtis distances with 95% confidence ellipses around age group (Adonis2 P = 0.08, F = 1.33).

The results of the PERMANOVA analysis performed on the Bray–Curtis distances using the adonis2 function revealed no significant differences in beta diversity between the four age groups (p = 0.08) (Fig. 5).

**Figure 6:**
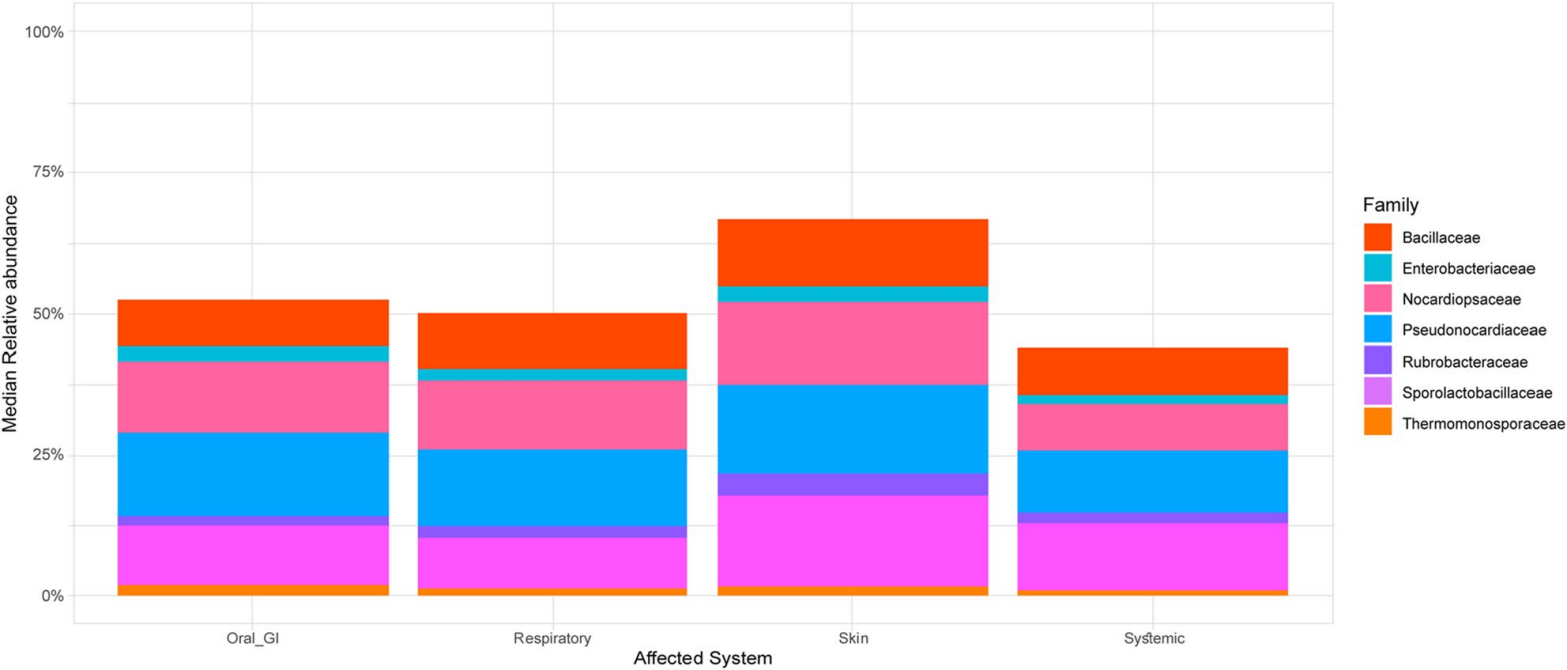
Taxonomic distribution of the febrile feline blood microbiota (*n* = 140) at family level by affected body system. Only families with a median relative abundance > 1% are included.

Although the pairwise Wilcoxon test revealed no significant differences at the phylum level for affected body systems in febrile cats, family-level comparisons yielded significant results via the Kruskal–Wallis rank sum test (p = 2.2E−16). Further, a pairwise Wilcoxon test of febrile cats by affected body system revealed no significant differences at the phylum level. It did, however, point out significant differences in family and genus composition, between almost all affected body system groups (Oral/Gastrointestinal(GI) *vs* Respiratory, p = 0.0027; Oral/GI *vs* Skin, p = 6.2e−5; Respiratory *vs* Skin, p = 2.4e−16; Respiratory *vs* Systemic, p = 6.1e−9; Skin *vs* Systemic, p = 0.00089), except for the family composition between systemic and Oral/ GI groups, which did not demonstrate significant differences (p = 0.122) (Fig. 6).

The top three families in the Oral/GI group by median relative abundance were *Pseudonocardiaceace* (14.8% [31.2–0.1%]), *Nocardiopsaceace* (12.7% [0.1–25.3%]) and *SporolactoBacillaceae* (10.6% [0.1–28.3%]). In the Respiratory group the top three families were *Pseudonocardiaceace* (14.2% [1.1–23.2%]), *Nocardiopsaceace* (12.6% [1.1–39.6%]), and *Bacillaceae* (10.3% [0.7–20.4%]). As for the Skin group, the top three were *SporolactoBacillaceae* (16.1% [4.1–27.9%]), *Pseudonocardiaceace* (15.7% [2.6–32.2%]), and *Nocardiopsaceace* (14.8% [2.9–38.5%]). Finally, the top three families in the Systemic group were *SporolactoBacillaceae* (11.2% [0.4–36.1%]), *Pseudonocardiaceace* (11.1% [0.4–30.1%]), and *Bacillaceae* (8.5% [0.4–28.2%]).

### Comparing the healthy and febrile groups

**Figure 7:**
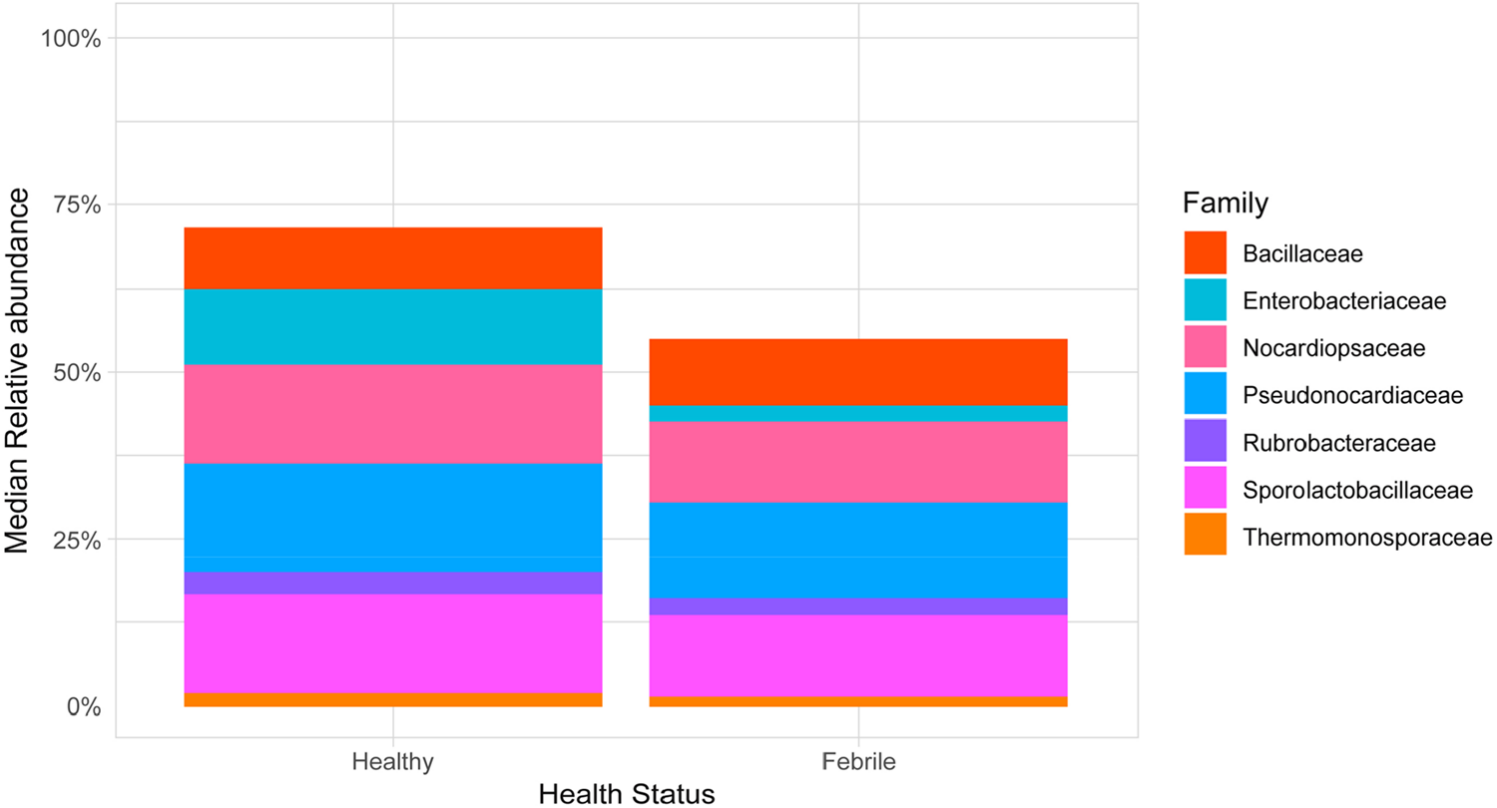
Significantly different (p = 0.0104) compositions of taxa between healthy and febrile cats, across all samples (*n* = 285) at the family level, which had a median relative abundance > 1%, in healthy (*n* = 145) and febrile (*n* = 140) cat samples.

**Figure 8:**
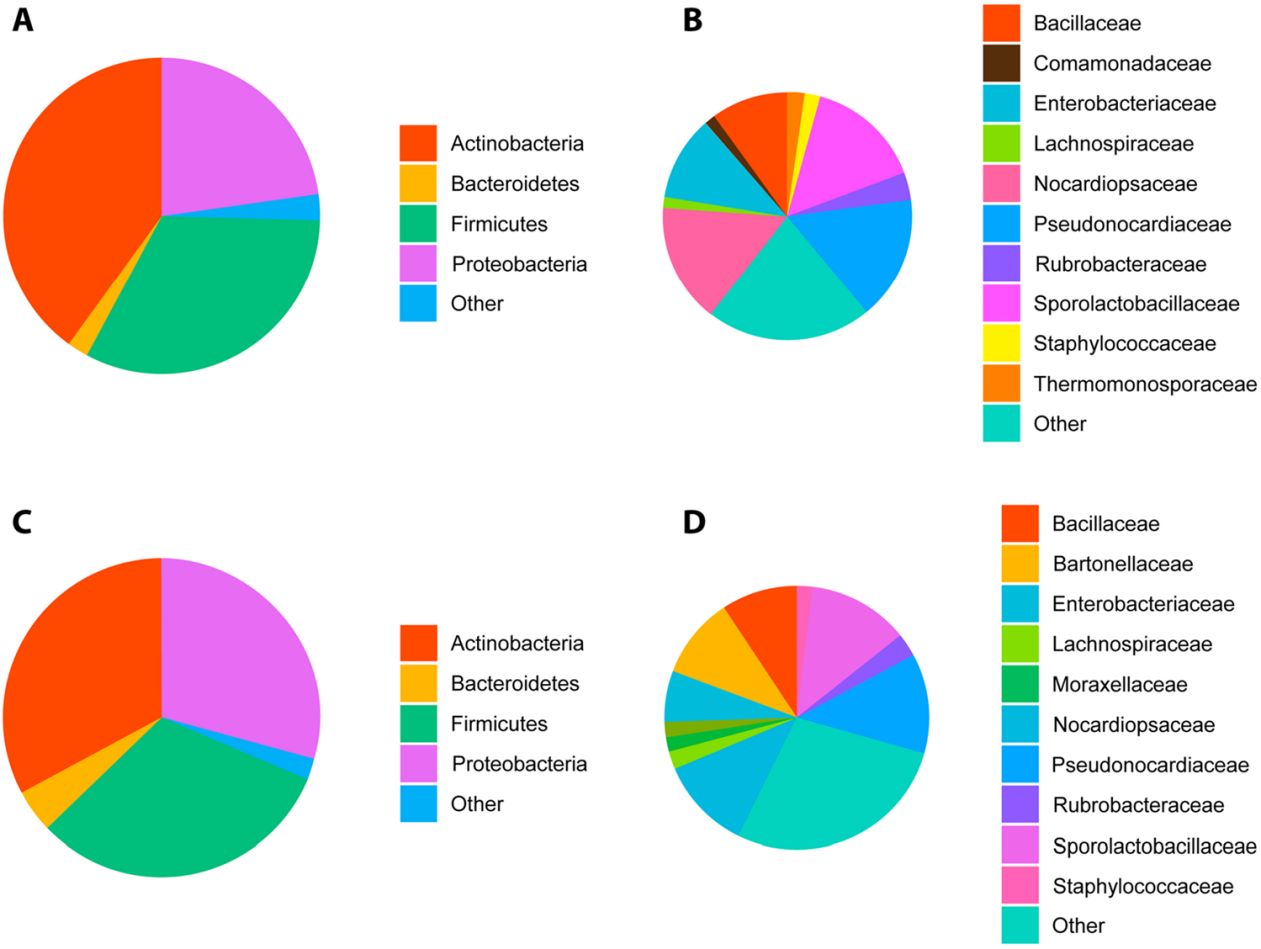
Pie charts representing the composition of healthy cats by phyla (**A**, greater than 1% median relative abundance) and family (**B**, top ten by median relative abundance), and febrile cats by phyla (**C**, greater than 1% median relative abundance) and family (**D**, top ten by median relative abundance). “Other” groups those phyla with a median relative abundance ≤ 1% or not in the top ten at family level.

While the Kruskal–Wallis rank sum test showed no differences in phyla composition, it did illustrate significant differences in family composition, between healthy and febrile cats (p = 0.0104; Figs. 7 and 8). For this analysis, families were filtered based on a median relative abundance > 1% of all the samples.

**Figure 9:**
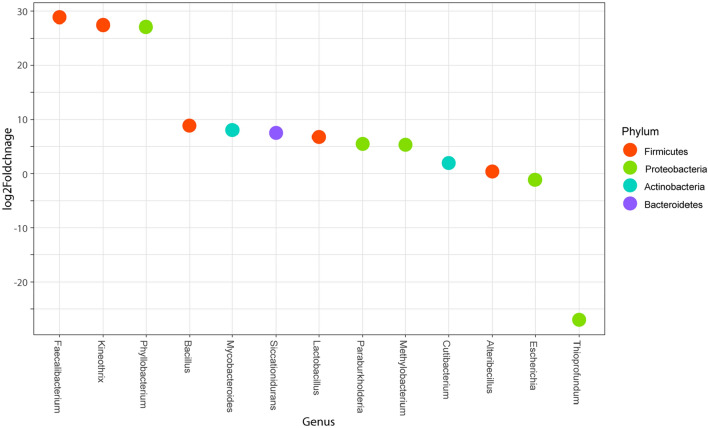
Differential abundance analysis (DESeq) assessing OTUs significantly changed (Padj < 0.05) in the blood microbiota of febrile cats (*n* = 140) compared with healthy cats (*n* = 145). Each circle represents significant OTUs at the genus level (x-axis), colored by Phylum. The positive values of log2 Fold Change (y-axis) indicate higher relative abundance in febrile cats and negative values show for higher relative abundance in healthy cats.

Testing for differential relative abundance between healthy and febrile cats revealed eleven phylotypes significantly increased in the febrile group (*n* = 140), and two phylotypes significantly increased in the healthy group (*n* = 145) (Fig. 9).

The most extreme of the log2fold changes belonged to the *Firmicutes* and *Proteobacteria* phyla. Specifically, the genera *Faecalibacterium* and *Kineothrix* (*Firmicutes*), and *Phyllobacterium* (*Proteobacteria*) experienced increased abundance (log2fold change > 25) in febrile samples. Whereas *Thioprofundum* (*Proteobacteria*) demonstrated a significant decrease in concentration (log2fold change < − 25).

Several diversity indices (Chao, Observed, Shannon, inverse Simpson) showed that alpha diversity did not differ significantly between healthy or febrile cats (Table 1).

**Table 1:** Summary statistics of alpha diversity indices (observed species; Chao1; Shannon; InvSimpson), calculated per group healthy (n = 144) and febrile cats (n = 140).

Diversity Index	Group	Min	Q1	Median	Mean	Q3	Max
Observed species	Healthy	6	16	20	24.3	29.5	65
	Febrile	9	14.8	20	31.8	30	397
Chao1	Healthy	6	16	20	25.1	30.2	102
	Febrile	9	14.8	20.5	37.4	31.1	666
Shannon	Healthy	0.491	2.06	2.25	2.35	2.61	3.56
	Febrile	0.148	1.96	2.27	2.34	2.62	4.75
InvSimpson	Healthy	1.28	6.00	6.96	8.48	9.59	26.2
	Febrile	1.05	5.57	7.40	10.1	9.83	73.9

**Figure 10:**
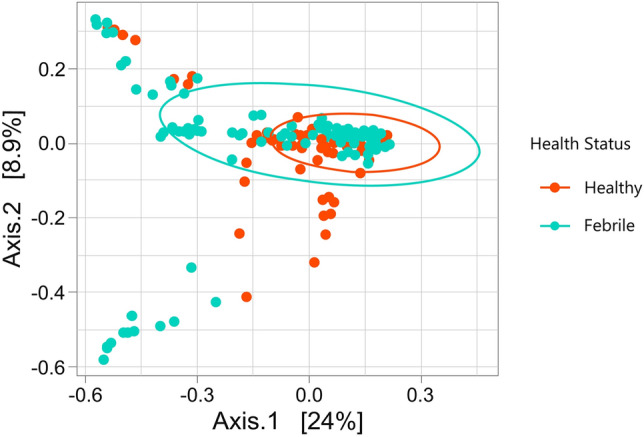
Principal Component Analysis (PCoA) of the blood microbiota of Healthy (*n* = 145) and Febrile cats (*n* = 140) using Bray–Curtis distances (R2 = 0.03, p < 0.01). Each data point represents an individual sample and ellipses represent a 95% confidence interval. The percentages in parenthesis are the proportion of variation explained by the PCoA axis.

The exception being between Shannon diversity indices (p = 0.039) and InvSimpson indices (p = 0.009) of all four age groups, across healthy and febrile cats (according to Kruskal–Wallis test). The mean Shannon diversity index for all cats was 2.33 ± 1.30. However, a PERMANOVA test based on Bray–Curtis distances showed that the beta diversity of the feline blood microbiota was influenced by the health status (febrile *vs* healthy) of the animal (PERMANOVA R-squared = 0.03042, F = 9.86, p = 0.000999) (Fig. 10).

**Figure 11:**
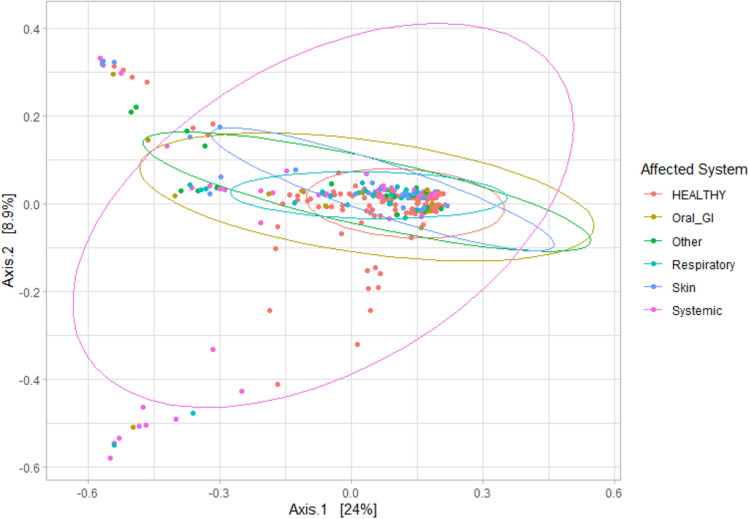
Principal component analysis (PCoA) visualization of febrile cats from the top four affected body system groups (*n* = 117; Systemic [*n* = 45]; Respiratory [*n* = 31]; Skin [*n* = 28]; Oral/Gastrointestinal [*n* = 15]), based on Bray–Curtis distances, with 95% confidence ellipses per group (Adonis2 P = 0.017, F = 2.07).

Bacterial community structure and phylogenetic diversity significantly differed when comparing febrile cats by affected body system with healthy cats, according to the Bray–Curtis beta diversity analysis, as distinct clusters could be observed (Adonis2 P = 0.001, F = 3.37; Fig. 11).

## Discussion

To the best of the authors’ knowledge, this is the first investigation exploring and comparing the blood bacterial microbiota between healthy cats and cats with fever. Furthermore, this is the first study of this magnitude (*n* = 145) to explore the blood microbiome of healthy cats. Whereas a previous investigation only looked at the blood microbiome of six healthy kittens^15^, the present work included 285 client-owned domestic cats (145 healthy, 140 febrile).

Fifteen samples from healthy cats failed to amplify any genetic material, this was expected since the blood microbiome is considered a lower-yield environment when compared to other well-studied sites^25,26^. This low-yield environment is largely caused by the inherent low bacterial biomass of the blood. Even so, next-generation sequencing allows this level of biomass to be characterized^27^.

In accordance with research in dogs^28^, the healthy blood microbiota of cats in this study was dominated at the phylum level by *Actinobacteria* (median relative abundance 39.33%) and *Firmicutes* (32.02%), as opposed to previous research in cats where it was dominated by *Bacteroidetes* (67.60%) and *Proteobacteria* (22.26%). In humans, the most abundant phyla were *Proteobacteria*, *Firmicutes*, *Actinobacteria*, and *Bacteroidetes*^7,29^. One of the many possible explanations for this discrepancy is that previous research analyzed samples from just six healthy, lab raised kittens (< 8 months old) originating from just two litters on the same diet^15^. As studies of the gut microbiome show, factors such as age, sex, environment, litter, and diet can all influence the gut microbiome^28,30^. Previous research comparing the gut and blood microbiota suggests that factors influencing the gut likely affect the blood as well^14^. Different methodologies or analyses could contribute to the differences between our two studies, such as differences in region of amplification in the 16S gene or in bio-informatics flow.

Phyla such as *Actinobacteria* great in number and diverse in niches occupied. These habitats include soil, rhizosphere, and marine and freshwater systems^31^. Therefore, relevance of taxa present is best inferred at the family level. The most abundant of which being: *Pseudonocardiaceae* (15.85%) which are aerobic, Gram-positive, and non-motile bacteria with low clinical significance. However, the genus *Pseudonocardia* had an important role in biotechnology due to its anti-bacterial, anti-fungal, and anti-tumor metabolites^32^; *Nocardiopsaceae* (15.26%), largely represented by the genus *Nocardia* which are nonmotile, non–spore-forming, pleomorphic gram-positive, facultative intracellular organisms. Feline nocardiosis is an opportunistic pathogen strongly associated with immunosuppressive disorders caused by Feline Leukemia Virus (FeLV) and Feline immunodeficiency virus (FIV)^33^. With a tendency to lie latent, it is not unbelievable to discover circulating evidence of this pathogen in healthy cats; *SporolactoBacillaceae* (14.73%) which are heat resistant and endospore forming rods. *SporolactoBacillaceae* are not clinically relevant, however their spores can be found in raw cows’ milk, as well as commercial probiotics^34^.

There were no significant differences in the alpha or beta diversity of the blood microbiota by age and sex within the healthy group. Therefore, these relative abundances could represent a degree of normality, or “eubiosis” in domestic cats^35^. This is in line with recent studies which found that individual variation had minimal effect when compared to stronger factors like disease and antibiotic use^36^. This “Anna Karenina principle” has been applied to animal microbiomes before, in which diseased or dysbiotic individuals vary more than healthy ones^37^. However, significant differences in the family composition between young adult and mature adult cats in healthy cats point to more distinct taxonomic makeups. Similar age-related associations have been noted in the canine gut microbiota and human blood microbiome^28,38^, but further research is required to elucidate that relationship.

It is evident that in the febrile group, the type of disease is a stronger driver than both sex and age, which did not express notable differences. Cats from each of the four groups (Systemic; Respiratory; Skin; Oral/Gastrointestinal) had different microbiota compositions when compared to the members of other groups. The development of these distinct, disease-specific taxonomic compositions is supported by research in humans^18,39,40^. Moreover, these specific states of dysbiosis can be of clinical relevance by helping to identify and perhaps mitigate effects of their respective diseases since the identification of potential biomarkers is considered a challenging task for many diseases. The blood microbiome analysis is considered a noninvasive approach to identifying disease biomarkers, and in the future may increase the accuracy of disease classification in cats and the efficacy of therapies. Nevertheless, many unresolved questions remain. The distinction between febrile and healthy cats at the lower taxonomic rank of families warrants further investigation of potential bacterial biomarkers in blood samples that are predictive of clinical syndromes. To this end, the data found in this study serve as a baseline reference for future studies on diagnostic testing in clinical settings.

The only body system groups that were not significantly different were the Systemic and Oral/Gastrointestinal groups. These results align with previous studies demonstrating a significant increase in *Proteobacteria* abundance in the blood of dogs afflicted with gastrointestinal disease^21,41^. It is noteworthy that *Proteobacteria*, as Gram-negative bacteria, are known producers of endotoxins, which could explain their heightened abundance in systemic conditions such as fever. Moreover, the association between similar microbiota compositions in systemic and gastrointestinal diseased cats, characterized by elevated *Proteobacteria* levels, provides a potential link to the "leaky gut" phenomenon, which is commonly observed in animals, specifically dogs and cats^41–43^. According to this theory, disruptions in gut barrier integrity can lead to increased permeability, allowing the translocation of bacteria and their byproducts into systemic circulation. *Proteobacteria*, being a common player in human disease and a notable endotoxin producer^44^, could contribute to the development of a "leaky gut" and subsequently facilitate the entry of their bacterial components, and a rise in relative abundance in the bloodstream.

The same three phyla dominated both the febrile and the healthy groups (*Actinobacteria*, followed by *Firmicutes* and *Proteobacteria*), with the febrile group demonstrating a slight (non-significant) increase in *Proteobacteria* (29.23% in febrile as compared to 24.01% in healthy). Also, within the febrile group, a 25 log2fold increase of the genus *Phyllobacterium* was appreciated. This change can be attributed to the negligible concentration of the genus in the healthy group (< 0.0001%), and then a still negligible, but slight increase in the febrile group (< 1%). *Phyllobacterium* is a group occurring in the roots of plants and has recently been associated as a gut microbial marker of disease in mice^45^. Also in the febrile group, the genera *Kineothrix* and *Faecalibacterium* experienced a dramatic increase in relative abundance (> 25-fold). Both *Kineothrix* and *Faecalibacterium* are well known beneficial gut bacteria, responsible for producing short chain fatty acids, such as butyrate which supports digestion and immune function. Furthermore, both genera of *Firmicutes* can be included in commercial probiotic solutions^46,47^. The presence of these beneficial gut bacteria in the blood of febrile cats further supports the translocation of bacteria from the gut to blood in states of disease.

Families which had > 1% relative median abundance accounted for 71.46% of the total reads of healthy cats, as opposed to febrile cats where they only accounted for 54.89%. This lower richness in healthy cats further supports a state of eubiosis where a few “healthy” bacteria dominate the blood, whereas in disease, dysbiosis occurs. These changes are in line with differences described in many of the studies comparing blood microbiomes in health and disease^10–12,16,18,21,22^.

One of the limitations of this study is the sampling design, that was cross-sectional. While a recent study shows that fecal microbiota compositions are relatively stable when examined longitudinally, further research would be needed to make the same claim in the blood^36^. Another limitation of this study is the relatively low number of animals in certain affected body systems. It is crucial for future research to investigate the effects of these specific disease states on the blood microbiota to gain a more comprehensive understanding of how various health conditions influence microbial communities. Furthermore, yet another important limitation of this study is the lack of control over the diet of each individual cat. As these were client-owned pets and not laboratory cats, they were being fed a variety of food types and quantities. Perhaps the most important example of this limitation, which requires attention in future studies, is the potential for difference in blood microbiota between kittens pre and post weaning. As mentioned previously, diet is a major driver in the composition of blood microbiota and the switch from maternally produced milk to externally created food products should reflect that^14^. Finally, one should be aware that 16s rRNA sequencing generates compositional datasets for which differential abundance analysis implies several assumptions that can be overly reductionist^48^. Additionally, in a clinical setting, 16S sequencing in conjunction with culture could be useful for identifying viable bacteria. does not immediately imply microbial viability, but simply bacterial DNA presence. Also, studies have concluded that 16s rRNA sequencing methods are far more effective at qualitative assessments of complex microbial communities and are only semi-quantitative in the absence of adjunct techniques such as culture or propidium monoazide (PMA) treatment during extraction^49^. In future studies, the aforementioned techniques will have to be employed to assess the viability of said microbes.

## Conclusions

This study represents the first investigation exploring and comparing the blood-bacterial microbiota between healthy cats and cats with fever. By examining a larger sample size than previous studies, this investigation provides novel insights into the composition and diversity of the feline blood microbiota. The results indicated that in both healthy and febrile groups, sex was not a significant factor of microbial composition or diversity. Age exhibited some influence on the blood bacterial microbiota only in healthy cats, at family level, particularly between young adult and mature adult cats. While no differences at phylum level were observed between healthy and febrile cats, significant differences were illustrated at the lower taxonomic rank of families. Moreover, febrile cats displayed distinct familial compositions and beta diversities based on the underlying diseased organ system. Overall, the findings suggest that health status, nature of disease and age are significant drivers of blood microbial diversity and composition in cats, but sex is not. Understanding the factors that contribute to these differences can provide insights into the dynamics of the feline blood microbiome throughout different life stages and can have implications for managing feline health and disease prevention. Furthermore, the data found in this study can serve as a baseline reference in the future, as next-generation sequencing techniques pave the way for the future of diagnostic testing.

## Methods

### Samples origin

This study used stored genomic DNA samples purified from blood of client-owned domestic cats obtained from an extramural grant (FONDECYT REGULAR 1191462, PI: Dr. Ananda Muller). All experimental protocols from the former study were approved by the IACUC (Approval number: 353/2019), and consent was obtained from cat owner's as well as respective authority of Veterinary Hospital. The use of DNA samples for the current study was also approved by the Ross University School of Veterinary Medicine IACUC (TSU12.9.21). All methods were carried out in accordance with relevant guidelines and regulations and reported in accordance with ARRIVE guidelines. A cross-sectional consecutive sampling of 300 domestic client-owned cats (healthy [*n* = 160] and febrile [*n* = 140]) that had presented to the Veterinary Hospital (September 2019 to January 2021) was performed. To avoid sample contamination, blood samples were collected from all 300 cats in a safe and sterile fashion. The fur was shaved, and sterile gloves were used. Aseptic preparation of the venipuncture site was performed, using povidone-iodine with 70% ethyl alcohol. Blood was drained from a cephalic vein using a sterile single-use pediatric blood collection set (multiple sample adapter), disposable syringe and pediatric tubes (Nipro). Samples were then stored in anticoagulant-EDTA tubes and frozen in – 80 °C until DNA extraction.

### Cats and inclusion criteria

#### Healthy cats (n = 160)

Cats which presented to the Veterinary Hospital for vaccination, spaying, or as blood donors were considered healthy based on physical examination (inspection, palpation, percussion, auscultation, temperature measuring). Any cat with abnormalities on physical examination was excluded. Since host-associated microbiomes can be rapidly altered by exposure to antibiotics^50^, cats receiving antibiotic therapy within 30 days prior to consultation, were also excluded. Additionally, FIV and FeLV status (qualitative chromatography immunoassay for FIV Ab/FeLV Ag in serum) were obtained for all cats (healthy and febrile), as well as testing for Hemotropic *Mycoplasma* spp., *Cytauxzoon felis*, was performed via conventional (c) PCR. For the complete blood count, the following parameters were analyzed: red blood cell (RBC), white blood cell (WBC) and platelet counts; hemoglobin concentration; packed red cell volume; mean corpuscular volume (MCV); and mean corpuscular hemoglobin concentration (MCHC). An automated hematology analyzer, KX-21N (Sysmex©, Japan), was used. The blood smears were stained with rapid staining (Hemacolor^®^, Merck) for a differential WBC count. Only cats which were negative for the aforementioned pathogens and had a normal CBC were included in the healthy group. Age and sex were recorded at the time of blood sampling.

The healthy group was composed of 160 healthy cats ranging from 2 months to 15 years (median age: 2 years 6 months) old, both males and females. The sex subgroups were composed by 50.6% (81/160) females and 49.4% (79/160) males, of all ages. Age subgroups were formed accordingly to the 2021 AAHA/AAFP Feline Life Stage Guidelines^51^, as follows: kitten (birth up to 1 year); young adult (1–6 years); mature adult (7–10 years); senior (> 10 years). The total number of cats per age category was 60 kittens (subdivided in: 2 months–6 months [*n* = 30] and 7 months–1 year [*n* = 30]), 60 young adults (subdivided in: > 1 year–3 years [*n* = 30] and 4–6 years [*n* = 30]), 30 mature adults and 10 seniors.

#### Febrile cats (n = 140)

Cats were included if they had a body temperature > 102.5 °F (39.2 °C) for at least 24 h and during physical examination independent of a specific diagnosis. Any cat treated with antibiotics within 30 days prior to the beginning of the study was excluded. Cats with elevated body temperature likely due to hyperthermia from excitement, seizures, or environmental factors^52^ were also excluded. Age and sex were documented. Initial or definitive diagnoses were recorded at the time of blood sampling and used for categorizing abnormalities by affected body system.

The febrile group was composed of 140 febrile cats ranging from 3 months to 19 years (median age: 3 years), both males (55%; *n* = 77) and females (30.7%; *n* = 43) (unknowns 14.2%; *n* = 20). Febrile subgroups are described below (Table 2), and the ones in bold (Systemic [*n* = 45]; Respiratory [*n* = 31]; Skin [*n* = 28]; Oral/Gastrointestinal [*n* = 15]) were used for the comparison between subgroups.

**Table 2:** Number of febrile cats initially categorized by affected body system.

Affected body system	Initial/confirmed diagnosis per body system	Number of cats
**Systemic**	**Reduced general condition/anorexia; reduced general condition/FeLV positive; Reduced general condition/FIV positive; faucitis/eosinophilic granuloma/FIV and FeLV positive; reduced general condition/dysphagia; reduced general condition/constipation; fever of unknown origin, pale mucous membranes; prostration/pale mucous membranes**	45
**Respiratory**	**Feline upper respiratory syndrome; chronic rhinitis; dyspnea; bronchitis; pharyngitis; gingivostomatitis/rhinitis; feline upper respiratory syndrome/gingivitis; tongue ulcers; gingivostomatitis**	31
**Skin**	**Pyoderma; abscess; bite wound; squamous cell carcinoma; dermatitis; alopecia; myiasis**	28
**Oral/gastrointestinal**	**Diarrhea; giardiasis; gastritis; gastroenteritis; vomiting**	15
Urinary/renal	Renal disease; cystitis; cystitis with kidney stones; urinary obstruction; feline lower urinary tract disease (FLUTD)	7
Systemic/lymphatic	Renal lymphoma; mediastinal lymphoma/pyothorax; multicentric lymphoma; chylothorax	5
Reproductive	Mastitis; vulvovaginal discharge	3
Ear/eye	Otitis media; hypopyon/glaucoma; otitis/conjuntivitis	3
Locomotor	Fracture on limb, traumatism	2
Systemic/skin	Eosinophilic granuloma/oral bleeding	1
Total cats		140

Only the highlighted groups were used for intrafebrile group comparisons (by body system or age). The remainder were used for descriptive analysis of the febrile group and comparison with the healthy group.

### DNA extraction/purification

Genomic DNA was obtained using a commercial DNA extraction kit (DNeasy Blood and Tissue Kit Protocol for Animal Tissues–QIAGEN), according to the manufacturer instructions. The integrity of each blood-extracted DNA was confirmed by an endogenous control Conventional PCR targeting the feline 28S rDNA (Peters et al. 2008)^53^, being positive for all 300 samples. Negative controls in the form of nuclease-free water (NFW) were utilized to ensure sterile collection methods. For every 30 samples, one NFW was used as template DNA, totaling 10 negative controls. None of said negative controls demonstrated any amplification. Purified DNA was stored at − 80 °C, until 16S rRNA gene amplicon sequencing.

### Metagenomic analyses: V3-V4 16S rRNA gene sequencing

All 300 cats’ genomic DNA samples were sent to Macrogen (Geumcheon-gu, Seoul, South Korea) for 16S rRNA gene sequencing using the Illumina MiSeq platform. At Macrogen, total DNA was assessed for its quantity (picogreen method using Victor 3 fluorometry) and quality (gel electrophoresis method). The variable V3-V4 region of the 16S rRNA gene was amplified generating PCR products with a length of ~ 460 bp. For library construction, the Illumina official guide for 16S rRNA gene sequencing library preparation was used as a reference. As such, metagenome amplicon sizes were verified by running on an Agilent Technologies 2100 Bioanalyzer using a DNA 1000 chip. Library quantity of DNA templates (ng/µl) was performed using qPCR according to the Illumina qPCR Quantification Protocol Guide. Any metagenome amplicon presenting a concentration higher than 0 ng/µl was submitted for the sequencing and library construction.

### Bioinformatic and statistical analyses

Paired‐end reads were assembled and quality filtering on the raw reads was performed under specific filtering conditions to obtain the high‐quality clean reads according to the QIIME2^54^ quality-controlled process. Sequences that were not consistent with the target amplicon size, contained any ambiguous base calls or long runs (> 8 bp) of holopolymers, or did not align with the correct 16S rRNA gene region using the 128 Silva 16S rRNA reference database^55^, were removed. Sequences were also evaluated for the presence of chimeras using UCHIME^56^ and removed subsequently.

Taxonomy was assigned using the Ribosomal Database Project (RDP) taxonomy database and sequences were binned into operational taxonomic units (OTUs) at a 3% dissimilarity level using a de novo approach with the nearest neighbor method. Normality testing was performed and a subsample from the main dataset was used for richness and diversity calculations to decrease bias caused by non-uniform sequencing depth and to normalize sequence numbers. All downstream analyses including statistical and graphical visualizations were carried out in RStudio^57^ using packages tidyverse, dplyr, vegan, ape, ggplot2, phyloseq, pairwiseAdonis, and microbiome.

The alpha diversity of each sample was estimated by richness indices (observed species, Chao1) as well as the Shannon and Inverse Simpson indices.

For beta diversity, principal coordinate analysis based on Bray–Curtis dissimilarity metrics and (un)weighted UniFrac distances were used to assess differences in blood bacterial composition between healthy and febrile cats and within each group (healthy by age and sex; febrile by age, sex and affected body system) and tested by non-parametric multivariate analysis of variance (PERMANOVA). Although a total of 10 affected body systems were present in febrile cats, only body systems containing 10 or more animals per group were used for within febrile group comparison, as the minimal sample size recommended for microbial 16S rRNA gene comparison is 10 individuals per group (Kelly et al. 2015)^58^.

Significant differences in the abundance of OTUs were identified using a multifactorial negative binomial generalized linear model (GLM), implemented in the R package DESeq2. Significantly different OTUs (P-value adjusted by false discovery rate [FDR] < 0.05) between healthy cats and febrile cats and within each group (healthy by age and sex; febrile by age, sex and affected body system) were determined using the Wald test for significance of GLM terms.

### Ethics approval

This study that samples originated from was IACUC approved (approval number 353/2019) and owners fulfilled an authorization allowing the inclusion of their cats in the project. The use of DNA samples for the Every Cat Grant was also approved by the Ross University School of Veterinary Medicine IACUC (TSU12.9.21).

### Supplementary Information

Supplementary Figure S1.

Supplementary Figure S2.

Supplementary Table S1.

Supplementary Legends.

## Data Availability

The datasets generated and analyzed during the current study are available in the NCBI SRA repository submission number: PRJNA1040256. Data has not been released yet and will be available upon manuscript publication.
