# Supplementary figures and images for: Characterizing the blood microbiota in healthy and febrile domestic cats via 16s rRNA sequencing
Source: Sci Rep. 2024 May 8;14:10584. doi: 10.1038/s41598-024-61023-4 (PMC11079020; doi:10.1038/s41598-024-61023-4)

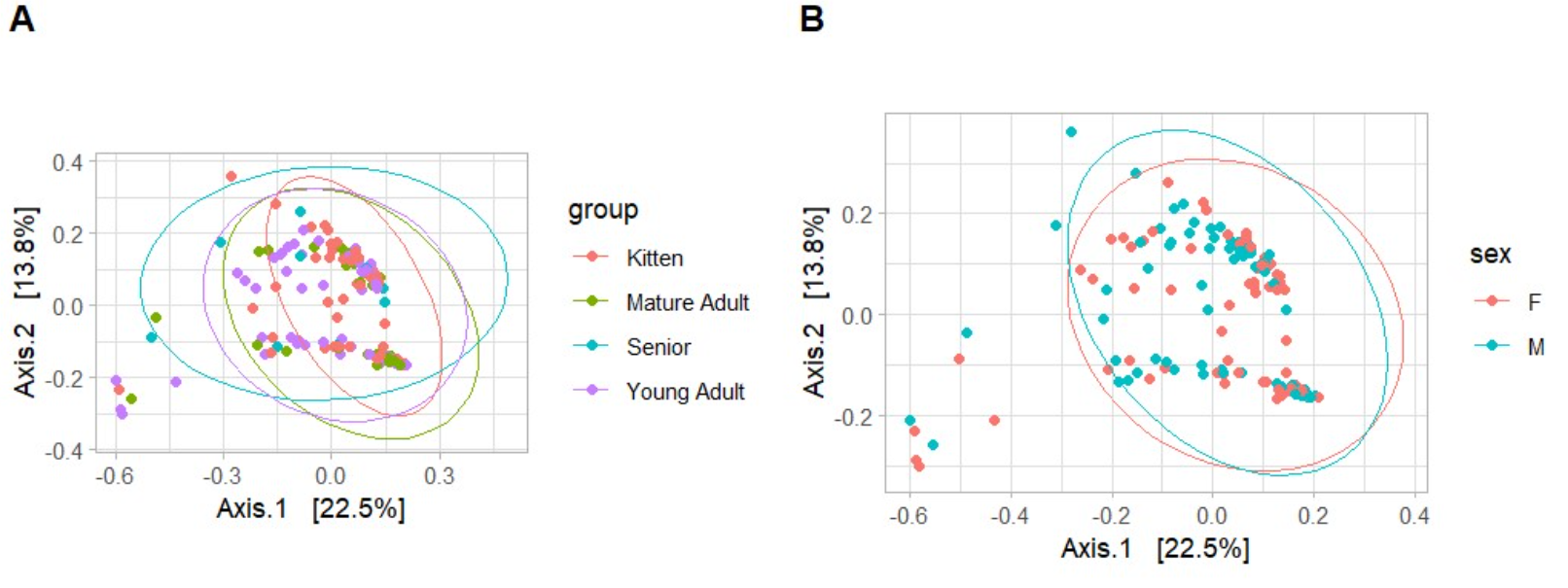

Supplement: Supplementary file 1 — Supplementary Figure S1. [file 41598_2024_61023_MOESM1_ESM.tiff]

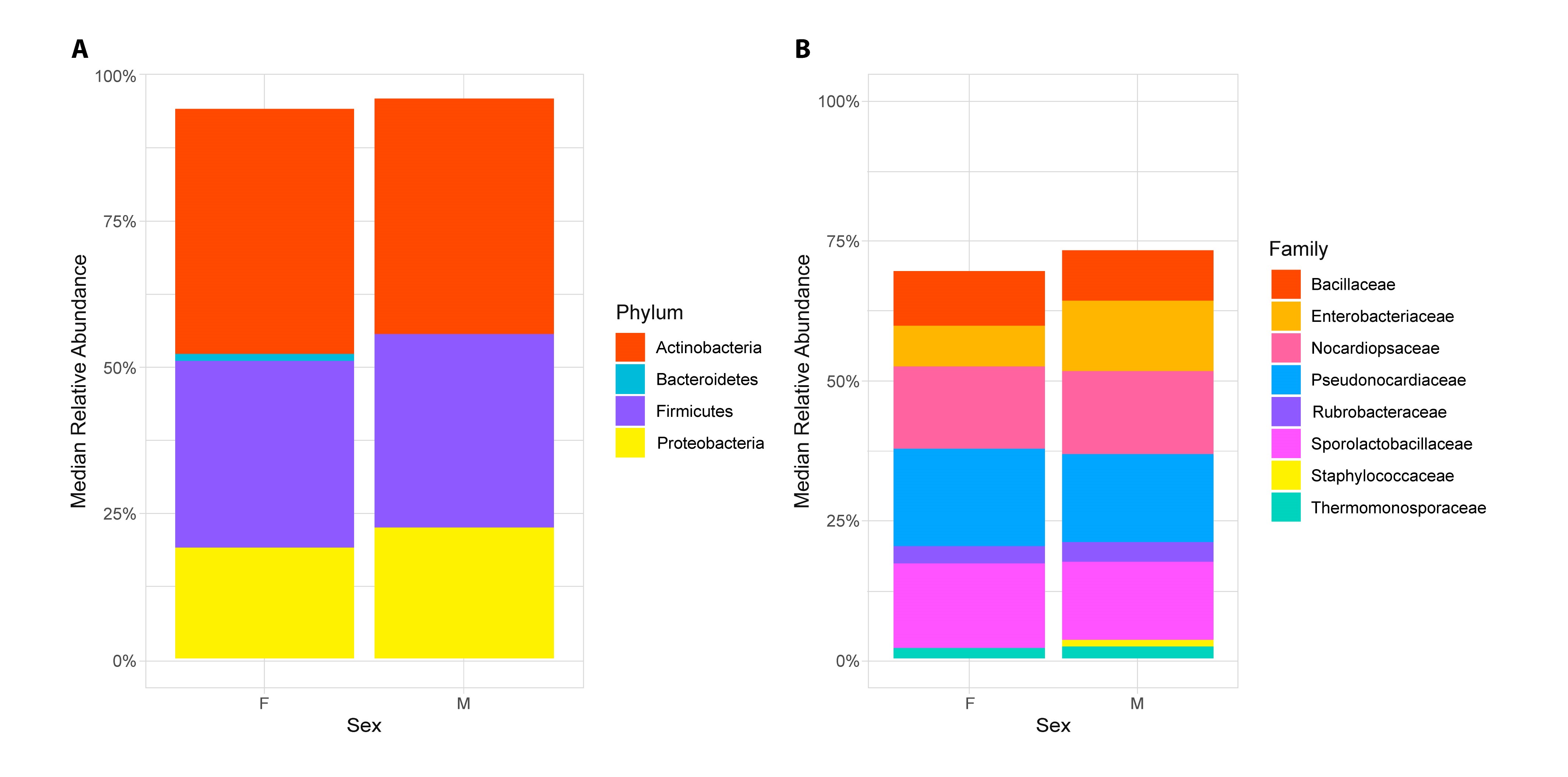

Supplement: Supplementary file 2 — Supplementary Figure S2. [file 41598_2024_61023_MOESM2_ESM.jpg]
