# Supplementary material for: Characterizing the blood microbiota in healthy and febrile domestic cats via 16s rRNA sequencing
Source: Sci Rep. 2024 May 8;14:10584. doi: 10.1038/s41598-024-61023-4 (PMC11079020; doi:10.1038/s41598-024-61023-4)
Supplement: Supplementary file 3 — Supplementary Table S1. [file 41598_2024_61023_MOESM3_ESM.docx]

**Table S1**. Number of healthy cats after exclusions, subdivided by age category.

| **Age Category** | | **Number of cats per age** | | |  |  |  |  |  |  |  |  | **Total/age** |
| --- | --- | --- | --- | --- | --- | --- | --- | --- | --- | --- | --- | --- | --- |
| Kitten up to 1Y | | 2M  (*n=*1) | 3M  (*n=*1) | 4M  (*n=*5) | 5M  (*n=*7) | 6M  (*n=*13) | 7M  (*n=*7) | 8M  (*n=*4) | 9M  (*n=*4) | 10M  (*n=*3) | 11M  (*n=*5) | 1Y  (*n=*5) | 55 |
| Young Adult 1-6Y | | >1 Y  (*n=*9) | 2Y  (*n=*9) | 3Y  (n =7) | 4Y  (*n=*10) | 5Y  (*n*=9) | 6Y  (*n=*9) |  |  |  |  |  | 53 |
| Mature adult 7-10Y | | 7 Y  (*n=* 11) | 8Y  (*n=* 9) | 9Y  (*n=* 6) | 10 Y  (*n=*1) |  |  |  |  |  |  |  | 27 |
| Senior >10Y+ |  | >10Y  (*n=* 10) |  |  |  |  |  |  |  |  |  |  | 10 |
| **Total Cats** |  |  |  |  |  |  |  |  |  |  |  |  | **145** |

*n=* number of cats; M=months; Y= Years
