# Supplementary material for: Characterizing the blood microbiota in healthy and febrile domestic cats via 16s rRNA sequencing
Source: Sci Rep. 2024 May 8;14:10584. doi: 10.1038/s41598-024-61023-4 (PMC11079020; doi:10.1038/s41598-024-61023-4)
Supplement: Supplementary file 4 — Supplementary Legends. [file 41598_2024_61023_MOESM4_ESM.docx]

**Figure S1**. Principal component analysis (PCoA) of the blood microbiota of healthy cats (*n=*142) excluding three outliers, with 95% confidence ellipses around age group (A, Adonis2 p=0.09, F=1.41) and sex (B, Adonis2 p=0.69, F=0.70).

**Figure S2**. Median relative abundances of operational taxonomic units (OTUs) at phylum (A) and family level (B) in healthy cats (*n=*145), by sex (M = male, F= female). Only these with median relative abundance >1% are included.
